# Dangerous Liaisons: Caspase-11 and Reactive Oxygen Species Crosstalk in Pathogen Elimination

**DOI:** 10.3390/ijms161023337

**Published:** 2015-09-28

**Authors:** JoAnn Simone Roberts, Ӧzlem Yilmaz

**Affiliations:** 1 Department of Oral Biology, University of Florida, Gainesville, FL 32610, USA; 2 Department of Periodontology, University of Florida, P.O. Box 100434, Gainesville, FL 32610, USA; 3 Emerging Pathogens Institute, University of Florida, P.O. Box 100434, Gainesville, FL 32610, USA

**Keywords:** caspase-11, caspase-4, caspase-5, ROS, autophagy, ATP, bacteria, infection, inflammasome, *Porphyromonas gingivalis*

## Abstract

Recently, the focus of murine caspase-11 and human orthologs caspase-4, -5 research has been on their novel function to induce noncanonical inflammasome activation in direct response to Gram-negative bacterial infection. On the other hand, a new role in anti-bacterial autophagy has been attributed to caspase-11, -4 and -5, which currently stands largely unexplored. In this review, we connect lately emerged evidence that suggests these caspases have a key role in anti-bacterial autophagy and discuss the growing implications of a danger molecule—extracellular ATP—and NADPH oxidase-mediated ROS generation as novel inducers of human caspase-4, -5 signaling during infection. We also highlight the adeptness of persistent pathogens like *Porphyromonas gingivalis*, a Gram-negative anaerobe and successful colonizer of oral mucosa, to potentially interfere with the activated caspase-4 pathway and autophagy. While, the ability of caspase-4, -5 to promote autophagolysosomal fusion is not well understood, the abundance of caspase-4 in skin and other mucosal epithelial cells implies an important role for caspase-4 in mucosal defense, supporting the view that caspase-4, -5 may play a non-redundant part in innate immunity. Thus, this review will join the currently disconnected cutting-edge research thereby proposing a working model for regulation of caspase-4, -5 in pathogen elimination via cellular-trafficking.

## 1. Introduction

The response towards an invading pathogen in the human body is part of a sophisticated system in innate immunity which involves a multitude of pro-inflammatory steps as a first line of defense. The initial steps involve the ability to sense “danger” signals such as microbial or host stress molecules via an array of recognition receptors (e.g., Pattern recognition receptors (PRRs), Toll-like receptors (TLRs), Nucleotide oligomerization domain (NOD)-like receptors (NLRs)) [1]. One platform that the immune system utilizes to trigger a pro-inflammatory response is called the inflammasome, a multimeric protein complex which forms in the cytosol in response to pathogenic microbes or danger signals, and triggers the secretion of mature pro-inflammatory cytokines, interleukin-1β (IL-1β) and interleukin-18 (IL-18) ultimately leading to a form of cell death called pyroptosis [1,2,3,4].

**Figure 1:**
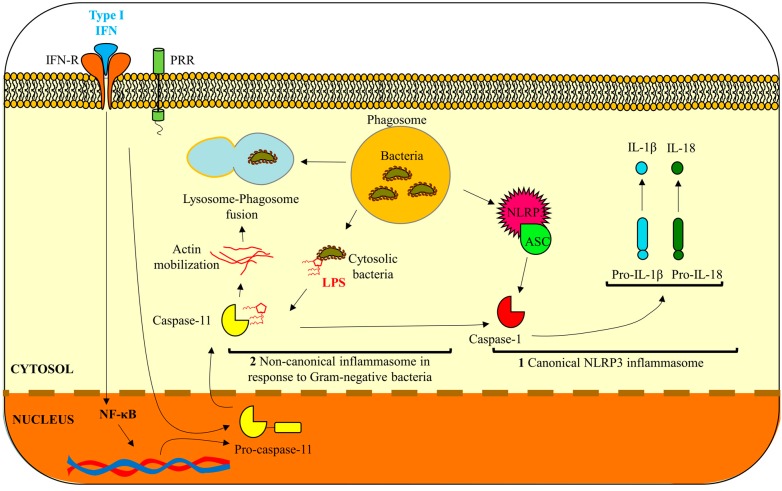
The role of caspase-11 in noncanonical inflammasome activation and anti-bacterial autophagy. 1: Activation of the NLRP3 inflammasome via caspase-1 leading to caspase-1 activation and an increase in the secretion of IL-1β and IL-18 [8,10,12]. Caspase-11 can form heterocomplexes with caspase-1 promoting the activation of NLRP3 inflammasome in response to Gram-negative bacterial infection [12]; 2: Caspase-11 promotes the destruction of the replicative niche of intracellular pathogens by promoting phagolysosomal fusion of pathogen-containing phagosomes through actin mobilization [6].

Inflammasomes, as described in multiple reviews, are grouped into canonical and noncanonical pathways [1,2,3,5,6]. A functional canonical inflammasome complex is typically composed of a nucleotide-binding domain leucine-rich repeat (NLR) protein, an adaptor molecule apoptosis-associated speck-like protein containing a CARD (ASC) domain, and caspase-1 [7]. The upstream regulators and specific molecules which form the inflammasome complex however depend on the danger signal/microbial inducer(s) [1]. For example, the most fully characterized NLR, pyrin domain containing 3 (NLRP3) inflammasome can be activated by extracellular ATP (eATP) danger signaling via the P2X_7_ receptor, reactive oxygen species (ROS) and/or from exposure to whole pathogens (Figure 1). In contrast, the AIM2 inflammasome is specifically activated by sensing double-stranded DNA in the cytosol and the interleukin-1β-converting enzyme (ICE) protease-activating factor (IPAF) inflammasome is activated by Gram-negative bacteria (e.g., *Salmonella typhimurium, Shigella flexneri, Legionella pneumophila,* and *Pseudomonas aeruginosa*) which have type III or IV secretion systems [1]. There are multiple inflammasomes, thoroughly reviewed in recent literature [8,9], which are activated by different distinct mechanisms yet converge to the maturation and release of pro-inflammatory cytokines IL-1β [8,9].

Over the past couple of years, special attention has been given to the cysteine-aspartic specific protease, murine caspase-11 (human orthologs caspase-4, -5) as a factor which drives noncanonical inflammasome activation [6,10,11,12,13]. Caspase-11 is classified as an inflammasome caspase grouped with human caspase-1, -4, -5, -12 (murine caspase-1, -11, -12), which are produced as monomeric zymogens that are then activated by dimerization and cleavage [14]. Once cleaved, these caspases can function as critical mediators of innate immunity in response to pathogenic stimuli [12,14]. Caspase-11 has become of specific interest in cellular microbiology due to its specific response to Gram-negative bacterial infection and novel ability to become activated from direct binding of the caspase activation and recruitment domain (CARD) to cytosolic lipopolysaccharide (LPS) via the lipid A moiety [12,15,16]. Caspase-11 binds and is activated by LPS with structural specificity to LPS containing six acyl groups, however species with four acyl groups, although able to bind, has not been shown to activate caspase-11 [17].

So far there are two cellular effects that have been described as result of caspase-11 activation. The first is the activation of the NLRP3 inflammasome leading to caspase-1 activation and an increase in the secretion of IL-1β and IL-18 [8,10,12]. Caspase-11 can form heterocomplexes with caspase-1 thereby inducing caspase-1-dependent pyroptosis in response to stress stimuli [12,18,19]. Second, Akhter *et al.* describes the ability of caspase-11 to destroy the replicative niche of intracellular pathogens by promoting fusion of the lysosome to pathogen-containing phagosomes [6]. This suggests a specific mechanism for caspase-11 in anti-bacterial autophagy independently of caspase-1 which may then lead to pyroptosis (Figure 1) [6,15,16]. The activation mechanisms and requirement of LPS binding for the promotion of phagolysosomal fusion by caspase-11 have not yet been fully studied.

Thus far, the focus of caspase-11 research has been solely on the ability of caspase-11 to induce noncanonical inflammasome activation, whereas its potential role in anti-bacterial autophagy has been otherwise ignored. Therefore, in this review, we examine murine caspase-11 and human orthologs caspase-4 and -5 as a potential regulator(s) of anti-bacterial autophagy by promoting phagolysosomal fusion in response to bacterial infection [6]. In addition, we discuss the potential implications of a danger signal, eATP, and ROS generation as novel inducers of human caspase-4 and -5 pathways during infection.

## 2. Inflammatory Murine Caspase-11

Caspase-11 was discovered based on its close similarity to caspase-1 and ability to induce pyroptosis [18]. The resting expression of caspase-11 is relatively low, with the exception of some epithelial cells [20,21], and is induced by detection of cytosolic LPS independently of the extracellular and vacuolar LPS receptor, Toll-like receptor 4 (TLR4) as repeatedly shown *in vitro* in macrophages, lymphocytes, hepatocytes, and epithelial cell studies [10,11,22,23,24] as well as *in vivo* [10,16,19]. The diversity of cell types in which caspase-11 functions is matched by the wide variety of pathogen infections it is able to protect the host from: *Burkholderia pseudomallei*, *Burkholderia thailandensis*, *S. typhimurium*, *Candida albicans*, *S. flexneri*, Enteropathogenic *Escherichia coli* and *L. pneumophila* [15,22,25,26,27,28].

Caspase-11 is transcribed from the same chromosomal locus as caspase-1 and localizes in the cytosol of the murine cell [18,19,22]. The expression and activation of caspase-11, in addition to direct binding with LPS, requires a priming signal by a pattern recognition receptor or damage-associated molecular pattern molecules (DAMPs) that induce transcriptional changes for inflammatory components [12,23,29]. Studies have shown evidence of IFN-β and IFN-γ in the activation of caspase-11 [29,30]. Moreover, the promoter region of caspase-11 contains several transcription factor binding sites including nuclear factor kappa-light-chain-enhancer of activated B cells (NF-κB) and signal transducer and activation of transcription 1 (STAT1)-binding sites [29]. The priming adjuvant for caspase-11 activation is then followed by a triggering signal (e.g., recognition of cytosolic LPS) to initiate the proteolytic cleavage of caspase-11 and cascade of irreversible cellular events such as pyroptosis/inflammation [12,23,29].

Until recently, the precise way of pyroptosis induction by caspase-11 has remained an unknown. Two recent novel studies have opened a window to understanding the specific mode of caspase-11 pyroptosis initiation [31,32]. The protein, gasdermin D (GSDMD), was identified as key molecule in LPS-stimulated pyroptosis by two genetic screens as described in Shi *et al.* [31] and Kayagaki *et al.* [32]. Both studies presented reproducible results indicating the cleavage of GSDMD is required for pyroptosis induction. Caspase-1, -4, -5, and -11 are all shown to cleave GSDMD; however caspase-11 was unable to cleave GSDMD unless first activated by LPS treatment. Therefore, GSDMD is proposed as a direct substrate of caspase-11 needed for pyroptosis induction [31,32]. These findings are the first depiction of a detailed action of caspase-11 in pyroptosis stimulation. Similarly, caspase-11 may have other, yet unidentified, substrates required for caspase-11 mediated anti-bacterial autophagy.

## 3. Are Human Caspase-4 and -5 Functional Orthologs of Murine Caspase-11?

As the function of murine caspase-11 especially in the immune response to Gram-negative bacterial infections becomes increasingly important, establishing functional conservation in human caspase-4 and -5 is critical to understanding this pathway as it relates to human systems. Currently, literature deriving from separate original studies suggests that human caspase-4 and -5 are functional orthologs of caspase-11. However, to date, there is still no clear answer.

As previously described, the ability of murine caspase-11 directly bind and sense cytosolic LPS is novel and unique as compared to other well-characterized inflammatory caspases in the activation of noncanonical inflammasome and pyroptosis [10,33]. Moreover, activation of caspase-11 requires a priming and triggering signal which can be attributed to LPS, IFN-β, IFN-γ, NF-κB, and STAT1 molecules [29,30]. Although majority of the studies of noncanonical inflammasome activation have been investigated in primary murine macrophages (caspase-11), evidence exists to support inflammasome regulation by human caspase-4 and -5 [34,35]. Human caspase-4 and -5 also bind directly to LPS and contain the same binding sites for upstream regulators NF-κB, STAT1 and IFN-γ [10,12,29,36], strongly suggesting a similar regulatory mechanisms between both murine and human caspases. In addition, the ability of human caspase-4 and -5 to induce pyroptosis in response to cytosolic LPS has been demonstrated *in vitro* [17]. Shi *et al.* demonstrated in macrophages, keratinocytes (HaCaT), and certain epithelial cells (HeLa, HT-29) the ability of caspase-4 to bind to cytosolic LPS and induce pyroptosis. Most intriguingly, caspase-4 was also able to replace caspase-11 function in *casp11* knockdown murine macrophages [17]. Similarly, Kajiwara *et al.* demonstrated the functional substitution capability of caspase-4 and -5 for caspase-11 in response to LPS when *casp11* is deleted *in vivo* [34]. Growing studies continue to demonstrate conserved function between human caspase-4, -5 and murine caspase-11 as shown in Shi *et al.* and Kayagaki *et al.* which describe caspase-4, -5’s ability to cleave GSDMD, a cytoplasmic protein required for LPS-induced pyroptosis [31,32].

Caspase-4 and -5 are believed to have come from a gene duplication event [37] and although recent studies show conserved functions; there is also some slight divergence between caspase-4 and -5 which have been observed. Caspase-5 was initially proposed to be the human ortholog of caspase-11 because of its similar expression pattern and sequence homology [38] whereas caspase-4 was not as well studied in earlier years [39]. However, recent examination has suggested that caspase-4 and -5 may play a non-redundant role in different cell types. For example, Casson *et al.* demonstrated an ability of caspase-4 to activate the inflammasome and induce pyroptosis in macrophages in response to *L. pneumophila*, *Yersinia pseudotuberculosis*, and *S. typhimurium* infection [10]. Caspase-5, however, although its expression was upregulated, was not cleaved and activated by the above pathogen infections. Another example was presented in a study by Knodler *et al.* which showed that in gut epithelial cells, caspase-4 expression was most abundant [11]. Furthermore, the maturation of pro-inflammatory cytokine IL-18 as a result of noncanonical inflammasome activation was prevented by siRNA knockdown of caspase-4 thereby enhancing the number of *S. typhimurium* which survived in the gut epithelium. However, knockdown of caspase-1 or caspase-5 did not have the same effect on inflammasome activation, IL-18 secretion or bacterial survival [11]. Based on the abundance of caspase-4 in keratinocytes and other epithelial cell types, it is tempting to speculate that caspase-4 has key importance in mucosal innate defenses and possibly in facilitating rapid signaling for the promotion of adaptive immunity [11,20,35].

Despite both caspase-4 and -5 possessing the ability to bind LPS [33,34], caspase-5 expression is inducible by LPS similar to caspase-11; however caspase-4 is constitutively expressed in human epithelial cells and is reported to be only modestly induced upon infection [11,18,34]. In contrast, studies in human primary macrophages show caspase-4 and not caspase-5 to be inducible by LPS [10]. These differences in LPS-driven induction provide further evidence that caspase-4 and -5 function may be cell-type specific. It also suggests potential additional mode(s) of regulation when we discuss the role of caspase-11, -4, and -5 in anti-bacterial autophagy (phagolysosomal fusion) [6], Currently, it has not been examined whether LPS is required for this function or if caspase-4 and -5 ability to promote fusion can be controlled by another regulatory mechanism.

Taken together, the current literature suggests that human orthologs caspase-4 and -5 function similarly to murine caspase-11 in the activation of the noncanonical inflammasome and induction of pyroptosis in response to infection and LPS detection. However, differences revealed in the studies describe that caspase-4 and -5 may vary in expression and response depending on the cell type or pathogenic stimuli [10,11,17]. Moreover, caspase-4 may have specific functions in mucosal immunity that caspase-5 has not yet been shown to have. Currently, there is no study that demonstrates caspase-4 and -5 working in conjunction, which suggests a non-redundant role for them in promoting immune response to Gram-negative bacteria.

## 4. Murine Caspase-11 and Anti-Bacterial Autophagy Systems: A Conserved Role in Humans?

**Figure 2:**
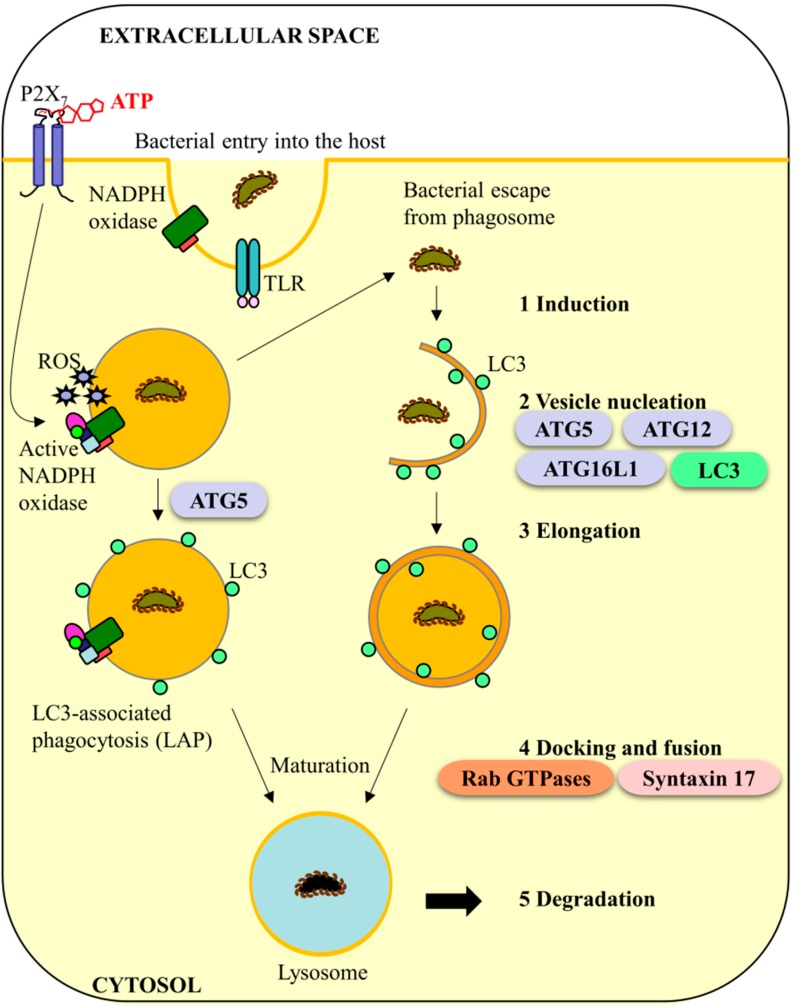
Progression of LC3-associated phagocytosis (LAP) and anti-bacterial autophagy in response to infection. **Left:** LAP begins by phagocytosis of the bacteria into the cell. Reactive oxygen species (ROS) generated by NADPH oxidase (NOX) as a result of extracellular ATP/P2X_7_ signaling can disturb the integrity of the phagosome resulting in either bacterial escape into the cytosol or recruitment of LC3 to the phagosome isolation membrane by ATG5 [41,42,43]. Attachment of LC3 to the membrane promotes rapid fusion with the lysosome resulting in microbial killing [44,46]; **Right:** Bacteria that escape the nascent phagosome can be targeted for autophagic clearance. Autophagy has five major steps: (1) induction; (2) vesicle nucleation; (3) elongation; (4) docking and fusion; and (5) degradation and recycling [44,46,48]. Initially, a phagophore surrounds the bacteria. This involves ATG proteins 5, 12 and 16L1 which initiate a cascade of conjugation steps to form a closed double-membrane organelle called an autophagosome [44,46,48]. The phagosome matures similar to LAP where it directly fuses with the lysosome to and degrades the autophagosomal contents together with the inner autophagosomal membrane [46]. The attachment of Syntaxin 17 allows for fusion to occur [49] while Rab GTPases are important determinants for each stage of maturation [44].

In innate immunity, LC3-associated phagocytosis (LAP) and autophagic clearance of invading bacteria are distinguished by the presence of a single or double-membrane vacuole formation [40]. Both LAP and autophagy pathways share similar upstream triggers (e.g., TLRs) and share some of the same machinery such as LC3, ATG5, and NADPH oxidase (NOX) which suggest that these two pathways may tightly collaborate in the host defense systems [41,42,43]. Moreover, some bacteria are able to escape the nascent phagosome by rupturing the membrane, which may result in the host response of forming the double-membrane autophagosome as a secondary clearance mechanism. In other instances, the phagosome membrane may undergo structural shifts thereby resulting in generation of reactive oxygen species (ROS) from NOX and thus more rapid clearance of the invading bacteria [44,45]. Regardless, the route, of bacterial killing, the final phagolysosomal fusion steps in both autophagy and LAP are similar (Figure 2) [44,46,47]. Therefore for the sake of importance, we will focus the remaining discussion on the described stages of autophagy and the relationship of caspase-11; however we do not negate that proposed mechanisms could also be attributed to LAP.

Autophagy generally functions to eliminate microorganisms, control pro-inflammatory signaling, regulate adaptive immunity and mediate secretion of immune regulators [41,46]. Autophagy has five major steps regulated by a variety of autophagy proteins: (1) induction; (2) vesicle nucleation; (3) elongation; (4) docking and fusion, and finally; and (5) degradation and recycling [44,46,48] (Figure 2). At the initial step, a phagophore is generated by cytoskeletal rearrangements by the membrane which surrounds an infectious agent. This involves ATG proteins 5, 12 and 16L1 undergoing a series of conjugation and cleavage steps to form a double-membrane organelle called an autophagosomes [44,46,48]. During maturation, the autophagosome directly fuses with the lysosome to form the autolysosome which degrades autophagosomal contents together with the inner autophagosomal membrane [41,46,49]. Fusion with the lysosome (step 4) is a late stage maturation event in anti-bacterial autophagy that is strictly regulated and requires the involvement of Rab-family GTPases which mediate the changes between phagosomes stages, tethering proteins such as soluble *N*-ethylmaleimide-sensitive factor attachment protein receptor (SNAREs) such as Syntaxin 17, pH changes in the intracellular compartment of the phagosomes, and lysosomal enzyme synthesis in order to degrade the intracellular cargo [41,44,46,49,50,51].

Bacteria which are contained in phagosomes are detected by caspase-11 mainly through interferon inducible guanylate binding proteins (GBDs) which can alter the phagosomal membrane integrity thus allowing for LPS to release into the cytosol [52,53]. Bacteria may also escape the phagosome and reside in the cytosol thereby exposing caspase-11 to LPS resulting in the activation of noncanonical inflammasome [24,54]. However, many bacteria have developed mechanisms to counteract different steps in the anti-bacterial autophagic pathway in order to acquire replicative niches, promote persistence, and minimize “danger” signals from the host [51,55,56]. For example, pathogens like *Coxiella burnetti*, *L. pneumophila*, *Staphylococcus aureus*, *Mycobacterium tuberculosis* and *Brucella abortus* are able to counteract autophagic pathways for their intracellular survival by retarding the phagosome maturation process, escaping the phagosome, forming separate non-autophagic ER-rich vacuoles, and modulating host GTPase activation thereby affecting cytoskeletal rearrangement associated with anti-bacterial autophagy [51,56,57]. Other pathogens have developed ways to utilize the autophagic system [55] and create a replicative niche within autophagosomes like successful oral pathogen, *Porphyromonas gingivalis* [58,59]. *P. gingivalis* is a Gram-negative anaerobe and effective colonizer of oral mucosa that can affluently persist in oral epi-mucosal cells and downregulate the epithelial immune response [58,60,61,62,63,64,65]. *P. gingivalis* survives predominantly inside ER-rich autophagosomes in primary gingival epithelial cells as well as endothelial cells either by preventing the fusion of autophagolysosomes or completely inhibiting normal autophagic flow [51,58,66,67]. In other instances, autophagy is able to effectively eliminate the invading pathogen as is the case with *Mycobacterium smegmatis*, *S. typhimurium*, and *B. pseudomallei* [51,68]. This describes a dynamic interplay between pathogen survival and autophagy machinery where autophagy targets bacteria for degradation and bacteria use that same host machinery to create an intracellular niche for survival [51,55,56].

Murine caspase-11 and human functional orthologs caspase-4 and -5 have been suggested to play an important role in the autophagic pathway having an independent function in controlling the fusion of pathogen-containing phagosomes with lysosomes via actin polymerization leading to restriction of *L. pneumophila* growth in macrophages [6]. Past this study there has not been much investigation into the role of caspase-11, -4, or -5 in bacterial killing specifically by phagolysomal fusion. However, a study on caspase-4 function during *S. typhimurium* infection in human intestinal epithelial cells revealed that *casp4* depletion enhanced cytosolic replication of *S. typhimurium*, but did not affect vacuolar replication [11]. This novel observation demonstrates the high level involvement of caspase-4 in restricting bacterial growth (*S. typhimurium*) and also suggests the ability of caspase-4 to activate host bacterial clearance mechanisms in response to cytosolic bacterial stimuli. There is some overlap between the upstream regulators of phagolysosomal fusion and regulators of caspase-4 activation which may also suggest a critical role for caspase-4 in promoting fusion of pathogen-containing phagosomes. For example, in macrophages, NF-κB controls phagolysosome fusion in the presence of *M. tuberculosis* infection [68]. Similarly, both human caspase-4, and -5 as well as murine caspase-11, expression and activation has been shown to be, in part, regulated by NF-κB [10,12,29,36]. Therefore, human caspase-4 and -5 may have an essential function in regulating phagolysosomal fusion, however additional studies are required to completely answer this question.

Furthermore, as other bacteria described before have the ability to counteract autophagic pathways, there is also evidence of the ability to inhibit caspase-4. *S. flexneri* possesses a caspase-inhibitor, bacterial effector OspC3, which blocks caspase-4 activation by binding in the catalytic pocket of caspase-4 [27] and *Francisella novicida* encodes an LPS that is not readily detectable in murine macrophages [10]. Another example is the oral pathogen that survives in ER-rich autophagosomes *P. gingivalis*, [51,58] which has heterogeneous expression of different lipid a moieties [69]. Using lipid A phosphatases to alter the lipid A composition of its expressed LPS, *P. gingivalis* may therefore manipulate the immune response by expressing an underacylated LPS [69,70,71] which can bind, but not activate caspase-11 (human caspase-4,-5) [70,72].

Although murine caspase-11 and human orthologs caspase-4, -5 have been poorly explored, it is our belief that the evidence described that suggests an importance for murine caspase-11 and human caspase-4 and -5 in the promotion of phagolysosomal fusion and bacterial killing. This function has not yet been investigated for caspase-5; however, it would be of great interest and value to examine this potentially novel function further. Considering that all other studies examining caspase-11, -4 and -5 before and after the 2012 Akhter *et al.* study [6] have focused on noncanonical inflammasome activation via detection of cytosolic LPS, we would like to suggest that there may be unexplored regulators of caspase-11 in addition to LPS that may promote specifically caspase function in mediating phagolysosomal fusion. This may provide additional explanation for the utilization of the caspase-11 pathway by other opportunistic microbes such as *C. albicans* that do not contain LPS [26].

## 5. Are eATP and ROS Important in Caspase-4/11-Mediated Bacterial Defense?

**Figure 3:**
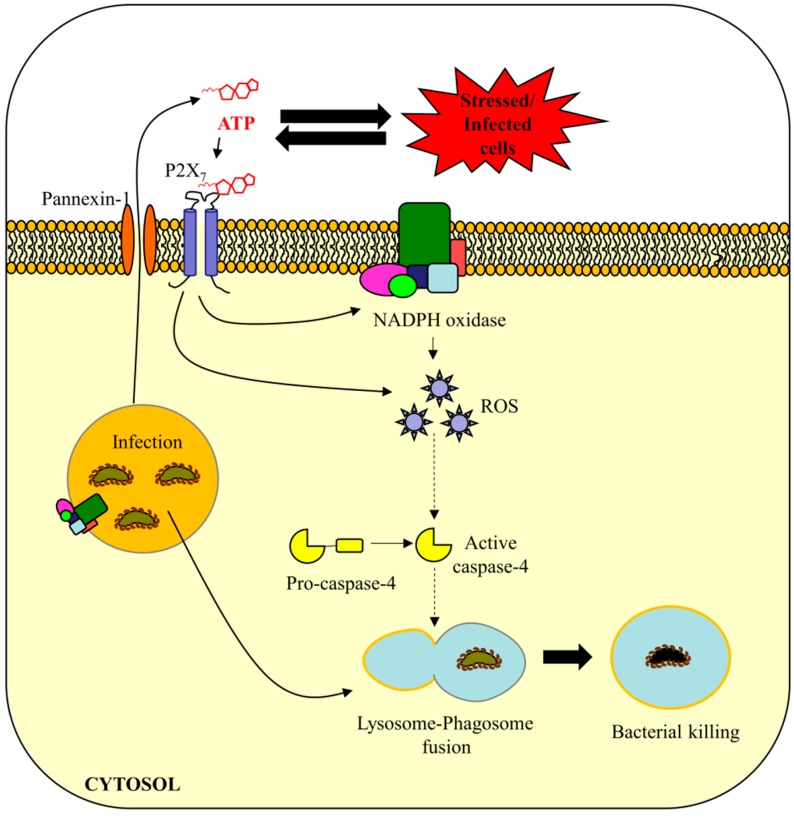
Proposed mechanism of caspase-4 activation in promotion of phagolysosomal fusion during pathogen infection. **Model:** Extracellular ATP (eATP) is an endogenous pro-inflammatory “danger” signal which is released from the host cell in response to stressed, injured, or infected cells [77,78]. eATP binds and activates the P2X_7_ receptor which results in calcium influx into the cell and potassium efflux [75,79]. Activation of this receptor promotes the formation of an active NADPH oxidase (NOX) on the plasma membrane/phagosome membrane [28,79,89]. The NOX-mediated reactive oxygen species (ROS) promotes the activation of caspase-4 which facilitates phagolysosomal fusion resulting in bacterial killing [89].

Currently the model for caspase-11 activation is that of a two-hit mechanism of a priming step and subsequent LPS recognition trigger of proteolytic cleavage [12]. Exploring the molecules involved in priming would give more insight into the regulatory mechanisms of the proposed anti-bacterial autophagy function (phagolysomal fusion) of caspase-11 specific for clearance of Gram-negative bacteria. Summarized below are potential connections made between eATP, ROS and anti-bacterial autophagy (Figure 3). The connections made are intended to incite interest into examining these pathways in response to Gram-negative infections and chronic infections across cell types, with the aim of better understanding the innate immune responses for therapeutic gains and insight in the field.

Pathogen infection usually results in host changes in ion fluxes, membrane damage, and ROS generation [28]. Over time some pathogens have developed the ability to circumvent the innate host defense systems such as autophagy, inflammasome, and ROS, through mimicry of human cell components, inhibition of pro-apoptotic and inflammatory pathways, or the formation of protective replicative niches within the host cell [51,56]. Many of these immune pathways have been studied in a variety of cell types because of the diversity of the pathogens able to invade cells at different sites in the body.

ROS is critically important in regulating antimicrobial defenses in the host cell and has a biocidal function at elevated levels [46,48,73]. Although there are multiple regulators of ROS, danger signal eATP is of specific interest in this review because of its significance in regulating immune responses to bacterial infections as well as its relationship to inflammasome activation and anti-bacterial autophagy [74,75,76]. eATP is an endogenous pro-inflammatory “danger” signal which is released from the host cell in response to cellular stress, injury, or infection [77,78]. Innate immune cells as well as epithelial cells express P2X receptors, in particular P2X_7_ and P2X_4_, which when bound to ATP, open calcium and potassium permeable ion channels that initiate a cascade of pro-inflammatory signaling modulators including ROS [75,79,80,81]. ATP activation of the P2X_7_ receptor further induces caspase-1-dependent inflammasome activation and subsequent IL-1β release [60,75,82,83]. Interestingly, the oral pathogen *P. gingivalis* is able to modulate eATP/P2X_7_ pathways including IL-1β production/release from primary epithelial cells by secretion of an effector, nucleoside-diphosphate-kinase (Ndk), to catalytically deplete eATP thereby modulating the ROS/oxidative stress and inhibit host cell death [60,62,63,65,84]. *P. gingivalis* modulation of ROS may promote its survival in ER-rich autophagosomes as the production of ROS coincides with initiation of autophagy in the epithelial cells [51,58,63,66,67]. Further evidence for the newly developing cross-talk between eATP, ROS, and anti-bacterial autophagy is described below.

Not surprisingly, in addition to canonical and noncanonical inflammasome, eATP-induced ROS production has been implicated in autophagy as well [28]. A study in 2008 in human macrophages showed that intracellular *Mycobacterium bovis* infections were cleared by eATP/P2X_7_-mediated phagolysosomal fusion [85]. Inhibition of the P2X_7_ receptor successfully blocked phagolysosomal fusion of *M. bovis*-containing phagosomes [85]. Another study in 2009 using microglial cells demonstrated the release of autolysosomes into the extracellular space as a pathogen clearance mechanism in response to the activation of the P2X_7_ receptor by eATP [86]. There are also regulators or products of caspase-11 activation which overlap with pro-autophagy machinery (e.g., IFN-γ, NLRPs, IL-1β) suggesting possible cross-talk between murine caspase-11 (human caspase-4 and -5) and eATP signaling [10,29,36,46].

A study in 2009 linked for the first time eATP to the activation of the NOX2 isoform in murine endotoxin-primed macrophages [79]. This however is poorly characterized and may be tissue-specific according to Meissner 2008 who showed murine peritoneal macrophages, which have distinct differences to adherent macrophages [87], did not have NOX2-specific ROS generation in response to eATP [88]. eATP has also been shown to have effect on the generation of ROS from other NOXs such as DUOX1 which generates peroxide (H_2_O_2_) in response to TLR ligands and produces pro-inflammatory cytokines [77]. The specific mechanisms, however, are also not currently known [77]. Therefore the specific isoform of NOX promoted by eATP signaling may be cell-type specific, however the generated downstream ROS has similar effects on pathogen killing [46]. Regardless, NOX-generated ROS has specific functions in regulating autophagy by targeting bacteria-containing phagosomes as described in Huang *et al.* [89]. This study demonstrated the importance of NOX2-generated ROS in the phagolysosome degradation pathway by recruiting critical autophagy proteins to the targeted *S. typhimurium*-containing phagosomes [48,89,90]. However, the specific mechanisms of this were not described. If we consider the independent function of caspase-11 in phagolysosomal fusion as described earlier [6] this would be an interesting path to explore in the activation of caspase-4 in humans. Another study identified an autophagy regulatory protein, RUBICON (run domain beclin 1-interacting and cysteine-rich-containing protein) that connects autophagy to NOX by positively regulating NOX-mediated ROS generation during microbial infection and promoting both phagocytosis and autophagy [47,91]. Therefore, studies have not yet concentrated in connecting eATP-induced ROS, NOX, and anti-bacterial autophagy pathways, however there is a myriad of strong implications of their importance in response to bacterial infection and potentially in regulating murine caspase-11 (human caspase-4 and -5) in phagolysosomal fusion.

## 6. Conclusions

Holistic review of the current literature strongly points to potential key connections between eATP, NOX-mediated ROS-generation and anti-bacterial autophagy (e.g., phagolysosomal fusion). The role of caspase-11 and human functional orthologs caspase-4 and -5 in activation of noncanonical inflammasome has been well described [12,23] and new information is continually being disseminated on this topic to the scientific community. However, the role of these special enzymes and immune regulators in the promotion of pathogen-containing phagosome fusion has been greatly over looked and key questions in the field remain to be answered. First, the role of human caspase-4 and -5 in phagolysosomal fusion has not been explored mechanistically beyond actin polymerization nor has it been investigated in other cell types beyond macrophages. This is a limitation in that bacteria with the capability to survive by circumventing autophagy machinery have been shown to infect a variety of hematopoietic cells as well as epithelial cell types as is this case with chronic opportunistic pathogen infections at mucosal surfaces [27,51,55,56,57,58,67]. Moreover, studies have also indicated that there may be deviating roles and expression for caspase-4 and -5 depending on the cell type and the microbial inducer [10,11]. Notably, the studies point to a specific importance for caspase-4 in mucosal immunity since caspase-4 expression level and ability to be activated in response to infection highly differs compared with caspase-5 [11,35]. Accordingly, caspase-4 may provide valuable insight into the rapid sensing and clearance of bacteria at mucosal tissues. Second, eATP regulation of NOX-generated ROS appears to have key significance in autophagy based on data suggesting recruitment of autophagy machinery to pathogen-containing phagosomes and regulation by identified autophagy proteins such as RUBICON [45,47,48,89,91]. The link between these upstream immune regulators is still largely unexplored territory. Examination of the importance of these pathways in the activation of novel murine caspase-11 (human caspase-4, -5) function in autophagic clearance of bacteria may reveal interesting mechanistic insights, predispositions and potential therapeutic strategies for Gram-negative infections in humans. As we have eluded before, there may be alternate regulation mechanisms of caspase-11, -4, and -5 in addition to LPS binding. The ability of non-bacterial pathogens such as *C. albicans* [26] to activate caspase-11 as well as the different cell-type expression levels of caspase-4 and -5 upon stimulation support this possibility [10,11,17,18,34]. Furthermore, it has not been investigated if LPS or LPS alone is necessary for the potential role of caspase-4, -5 in promoting phagolysosomal fusion in anti-bacterial autophagy [6]. It may be of great interest to investigate other activation mechanisms of these inflammatory caspases to facilitate bacterial killing in host cells. In particular, bacterial pathogens with the potential ability to avoid bacterial clearance by phagolysosomal fusion or to inhibit caspase-4 such as *P. gingivalis, M. tuberculosis, S. flexneri* and *F. novicida,* would be prime candidates for future studies [10,27,51,70,71]. Hence, exploration into human caspase-4 and -5 function and regulatory mechanisms will provide critically novel insight into the regulation of human innate immune responses to pathogen infection at epi-mucosal sites and in immune cells. The information gained may provide novel treatment targets for Gram-negative bacterial infections and other pathogens that are able to direct host machinery promoting pathogen survival and persistence.
